# Low-Temperature, Solution-Processed, Transparent Zinc Oxide-Based Thin-Film Transistors for Sensing Various Solvents

**DOI:** 10.3390/ma10030234

**Published:** 2017-02-26

**Authors:** Hsin-Chiang You, Cheng-Jyun Wang

**Affiliations:** Department of Electronic Engineering, National Chin-Yi University of Technology, No. 57, Section 2, Zhongshan Road, Taiping District, Taichung 41170, Taiwan; eddie415004@gmail.com

**Keywords:** thin-film transistor (TFT), zinc oxide (ZnO), bio-sensor, transparent conducting oxide (TCO), polar solvent

## Abstract

A low temperature solution-processed thin-film transistor (TFT) using zinc oxide (ZnO) film as an exposed sensing semiconductor channel was fabricated to detect and identify various solution solvents. The TFT devices would offer applications for low-cost, rapid and highly compatible water-soluble detection and could replace conventional silicon field effect transistors (FETs) as bio-sensors. In this work, we demonstrate the utility of the TFT ZnO channel to sense various liquids, such as polar solvents (ethanol), non-polar solvents (toluene) and deionized (DI) water, which were dropped and adsorbed onto the channel. It is discussed how different dielectric constants of polar/non-polar solvents and DI water were associated with various charge transport properties, demonstrating the main detection mechanisms of the thin-film transistor.

## 1. Introduction

In recent years, great progress has been made in biotechnology electronic devices which are a great aid in human health and longevity research. Fluorescence techniques have been mainly used for detecting biological diseases traditionally, but the detecting procedure is time-consuming and expensive. Among bio-electronic devices, thin-film transistors (TFTs) have much promise as novel tools to study biochemical interactions. They may be used in sophisticated medical devices as well as disposable electronic devices. Due to the maturation of TFT fabrication technology, small-size TFTs can be integrated into high-density TFT arrays for direct, rapid, and inexpensive medical diagnosis. Moreover, TFTs are based on transparent conducting oxide (TCO) materials and can be fabricated by solution-process based technique, showing some advantages of low cost and low-processing temperature and compatibility with flexible substrates [1,2,3,4]. Solution-processed semiconductors have been intensively investigated for use in large-area flexible sol-gel fabrication and electronic device inkjet-printing [5,6,7,8], However, it has remained a challenge to achieve high performance and stability of oxide semiconductors using low-temperature process in the range of 200 and 300 °C which is needed for flexible substrates. These substrates are especially interesting for biological applications and enable the use of a wide range of biocompatible and biodegradable materials and devices, including bio-inspired peptide nanostructures [9], 2D nanostructuring of biosensor with nanodiamonds [10], ion-sensitive electrochemical transistors [11], pH sensors [12,13,14], and various label-free biosensing thin-film transistors [15,16,17,18].

Herein, we report the design, evaluation and fabrication of solution-processed ZnO-based TFTs by low-temperature processing and some effects of polar and non-polar liquid solvents on the exposed ZnO channel.

## 2. Materials and Experimental Section

**Figure 1:**
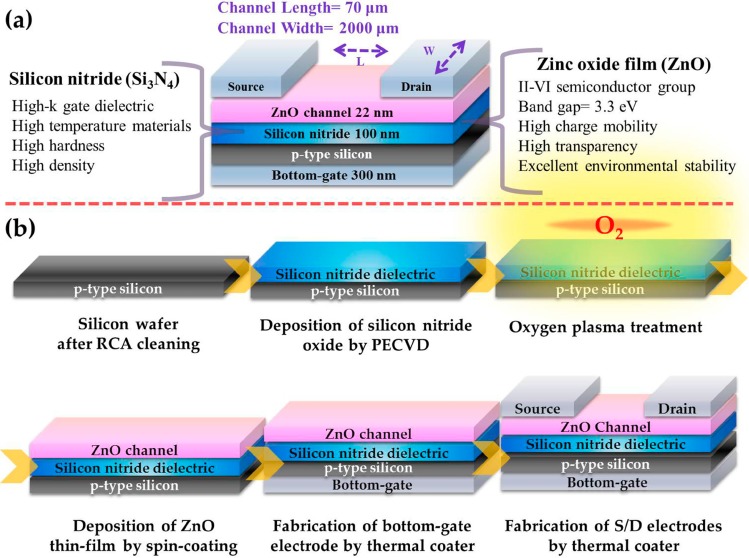
Zinc oxide (ZnO) based thin-film transistor (TFT) and its fabrication process. (**a**) Schematic diagram and illustration of ZnO-based TFT. For the TFT sensing, the ZnO film between two electrodes (source and drain electrodes, S/D) are exposed to various liquid solvents; (**b**) The fabrication procedure of a ZnO-based TFT by a low-temperature process.

Figure 1a presents the structure diagram of a zinc oxide based thin-film transistor (ZnO-based TFT) which is composed of bottom gate electrode, p-type silicon, silicon nitride, ZnO thin film, source and drain electrodes. The ZnO thin film has an exposed channel of 70 μm long and 2000 μm wide for sensing various non-polar and polar solvents. The film is fabricated using a 0.05 M ZnO solution precursor which is synthesized by dissolving zinc acetate dihydrate [Zn(CH_3_COO)_2_·2H_2_O] into ethanol (CH_3_CH_2_OH, absolute ≥ 99.8%) and stirring for 2 h at 60 °C.

Figure 1b shows the whole fabrication procedure of the TFT. First, a p-type silicon wafer doped heavily with boron (B) was treated via the standard Radio Corporation of America (RCA) cleaning process to remove metallic and organic contamination. Then, a 100-nm-thick silicon nitride (Si_3_N_4_) dielectric layer was deposited on the silicon substrate at 300 °C by plasma-enhanced chemical vapor deposition (PECVD). Subsequently, the ZnO precursor sol-gel solution was spin-coated on the silicon nitride dielectric layer. To form an active n-type semiconductor layer, spin-coating was carried out for 5 times at a maximum speed of 3000 rpm for 30 s, following an annealing process on a hot plate at 300 °C for 1 h. Finally, the bottom gate electrode was deposited on the back side of silicon wafer and the source and drain electrodes on the ZnO thin film by the thermal coating of 300-nm-thick aluminum (Al). A metal-mask of 70 μm long and 2000 μm wide was used during the deposition of source and drain electrodes to form an exposed channel on the thin film transistor.

## 3. Results and Discussion

### 3.1. Physical Analytics and Electrical Characterizations

**Figure 2:**
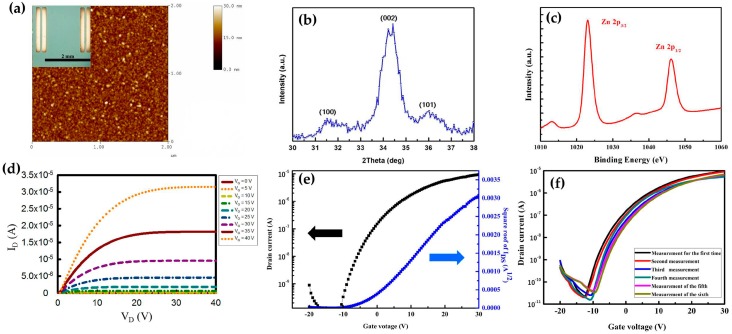
Physical analysis and electrical characterizations of ZnO-based TFTs in a dry environment: (**a**) Atomic force microscope (AFM) and the optical microscope (OM) image (inset) of the TFT; (**b**) X-ray diffraction (XDR) spectrum in the range of 30°–38°; (**c**) The X-ray photoelectron (XPS) spectrum of ZnO film showing Zn 2p peaks; (**d**–**f**) The electrical characterizations of the TFT; (**d**) I_DS_-V_DS_ curves, drain current as a function of drain voltage with the bottom gate voltage (V_GS,bottom_) varying from 0 to 40 V with steps of 5 V; (**e**) I_DS_-V_GS,_ drain current versus bottom gate voltage (V_GS,bottom_) varying from −20 to 30 V with steps of 500 mV, with the drain voltage being fixed at 5 V and the drain current being plotted on a logarithmic scale (left) and the square root of drain current plotted on a linear scale (right); (**f**) The endurance test for 10 iterations of I_DS_-V_GS_ curves.

Different techniques were used to characterize the ZnO-based TFTs. Figure 2a depicts a typical atomic force microscope (AFM) image of the ZnO thin film, indicating that the ZnO thin-film is continuous and smooth on the dielectric layer and the film roughness is less than 3 nm. Figure 2b shows the X-ray diffraction (XRD) pattern of the thermally annealed ZnO thin film.The major diffraction peak located at approximately 34.5° can be indexed to 002 of ZnO. This means that the grains in ZnO thin film may be textured with their *c*-axes along the film normal. The chemical structure and composition of as-deposited ZnO thin films were examined using X-ray photoelectron spectroscopy (XPS). The XPS spectrum (Figure 2c) shows characteristic peaks at 1023.1 eV and 1048.9 eV, which correspond to Zn 2p_3/2_ and Zn 2p_1/2_, respectively.

The electrical transport properties of the ZnO-based TFTs were investigated. Figure 2d shows the representative transfer characteristics of the devices in a dry environment, where the drain-source voltage (V_DS_) varies from 0 to 40 V and bottom-gate bias voltage (V_GS_) to the silicon substrate is in the range of 0 to 30 V at intervals of 5 V. The output drain-current (I_DS_) is controlled by both V_DS_ and V_GS_. These curves demonstrate saturation behavior of I_DS_ at high V_DS_ and saturation values of I_DS_ increase with V_GS_.

The transfer characteristics of the ZnO-based TFT are shown in (Figure 2e, black curve, left scale). The source-drain current I_DS_ on a logarithmic scale varies with the gate voltage V_GS_ (from −20 to 30 V) at a constant V_DS_ of 5 V. When V_GS_ was at 30 V, the I_DS_ is about 1.12 × 10^−5^ A in logarithmic scale indicating that the on-off current (I_ON_-I_OFF_) ratio was approximately 10^6^. (Figure 2e, blue curve, right scale) presents the square root of I_DS_ saturation values (I_Drain,sat_) vs. V_GS_. From I_Drain,sat_ vs. V_GS_ curves, the threshold voltage (V_th_) of 2.75 ± 2.86 V for the Si_3_N_4_ dielectric layer and the field-effect mobility (µ_FE_) of 0.0053 ± 0.0012 cm^2^·V^−1^·s^−1^ which were indicated by 0.0057, 0.0036, short circuit and 0.0065 can be determined according to the I_Drain,sat_ Equation (1), and the other derived parameters are summarized in Table 1. The errors were calculated on the basis of measurements on 4 TFT devices. The TFT device is turned on by the electrons accumulated in the n-type ZnO channel layer when V_GS_ is in the positive voltage direction, so the device operates in an “n-channel semiconductor enhancement mode”. As indicated by the linear plot, the I_ON_ value measured when V_Gate_ = 30 V and V_Drain_ = 5 V was 3.3 mA. The solution-processed ZnO-based TFTs not only show excellent electrical characteristics, but also good stability.

The electrical stability of a ZnO-based TFT was investigated by measuring the I_DS_-V_GS_ curves for 10 times on the same device, as shown in Figure 2f. The endurance test results of the TFT are repeatable and stable after 10 operations, so the TFT is suitable for sensing various liquid solvents.

### 3.2. Specific Detection of Polar and Non-Polar Liquid Solvents 

Liquid solvents can be generally divided into nonpolar molecule (e.g., toluene) and polar molecule (e.g., ethanol) categories because of high or low dielectric constants. In this section, we investigate the electrical effect of a nonpolar solvent (toluene), a polar solvent (ethanol) and water (deionized water) onto the exposed ZnO channel of the TFTs. All of the three solutions resulted in an immediate response of the TFT device when they were dropped onto the ZnO surface. Whether their electrical parameters gradually return to their initial states after solution degradations (drying) was also examined.

**Figure 3:**
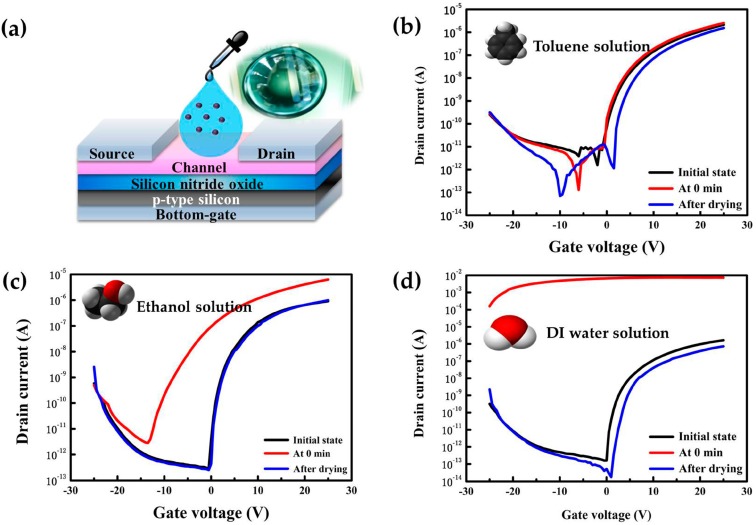
Transfer characteristics I_DS_-V_GS_ of solution-processed ZnO-based TFT sensors for various polar and non-polar solvents and DI water. (**a**) Illustration of dropping various liquid solvents on the ZnO sensing area between two electrodes (S/D); (**b**) A non-polar liquid solvent of toluene with a dielectric constant value of approximately 2.4; (**c**) A polar liquid solvent of ethanol with a dielectric constant value of approximately 24; and (**d**) A DI water with high dielectric constant of approximately 80, leading to a highly charged surface on the channel surface. The thickness of ZnO channel film used was approximately 22 nm prepared by the spin-coating shown in Figure S4.

The I_DS_-V_GS_ characteristics of the TFTs exposed to three liquids with different dielectric constant values are shown in Figure 3. Figure 3a shows the experimental diagram of the TFT device and liquid. All TFTs were operated in a saturated region with the V_GS_ varying from −25 to 25 V at a constant V_DS_ of 5 V. The “initial state”, shown in Figure 3b, means that the ZnO-based TFT device was not exposed to any solvents. When toluene was dropped onto the ZnO surface at 0 min, a threshold voltage (V_th_) did not show any significant negative shift (Figure S1) and no field-effect mobility (µ_FE_) degradation was observed. In contrast, the polar molecules were charged together on the interface of the ZnO film channel through the bottom-gate voltage, which determined the effective channel surface potential of the dielectric. From the drain current shown as the function of V_GS_**,** we observed a significant increase in the drain current (Figure 3c) when ethanol was dropped on the ZnO channel. Meanwhile, the V_th_ was shifted to a negative direction by about −6 V (Figure S2). Ionized water caused electrical shorts (Figure 3d and Figure S3) when it adsorbed on the ZnO channel at 0 min. toluene, ethanol and de-ionized water have dielectric constants of about 2.4, 24 and 80, respectively. The different response of the TFT to toluene, ethanol and de-ionized water may be related to their dielectric constants. The electrical parameters are summarized in Table 1. This V_th_ shift in the negative direction or electrical short was attributed to the extra electrons from the polar molecules accumulating on the ZnO channel due to their polarity or electronegativity. When the molecules are dropped and adsorbed on the exposed ZnO channel surface, a low V_GS_ operating voltage was required to turn on the TFT devices. Due to this phenomenon, the magnitudes of the V_th_ and V_on_ shifts depend on the dielectric constants of the solvents, leading to the successful demonstration of a solution-processed ZnO-based TFT solvent molecule sensing.

Those V_th_ and V_on_ values were almost recovered to their initial states via drying process. In addition to being used as a liquid molecule sensor, this solution-processed ZnO-based TFT device is a potential candidate for utilization as a bio-sensor in which its reaction with liquid solvents in bio-materials may lead to additional carrier charge on the interface between the semiconductor channel and biomaterials. Table 1 summarizes the electrical parameters of solution-processed ZnO-based TFT devices such as μ_FE_ and V_th_ for different solvents at the initial state, 0 min after droplet application, and after drying with respect to the dielectric constant of each solvent.

Drain current saturation equation:

I_Drain,sat_ = (W/2L)·C_i_·µ_FE_·(V_GS,bottom_ − V_th_)^2^ (1)

where W is the channel width, L is the channel length, C_i_ is the gate oxide capacitance per unit area, and C_i_ = (ε_0_·ε_r_)/thickness (F/m), ε_r_:Si_3_N_4_ = 6~7, µ_FE_ is the effective electron mobility.

## 4. Conclusions

In summary, we have demonstrated a solution-processed ZnO-based TFT sensor with a highly transparent semiconducting channel which is able to sense various polar and non-polar liquid solvents and DI water. The sensor is fabricated by a low-temperature (200–300 °C) processing technique. ZnO-based TFTs provide stable electrostatic operating voltages through bottom gate electrode control and a high I_ON_/I_OFF_ switching characteristic of approximately 10^7^. The ZnO-based TFT sensors are of low cost and environmentally safe, which is imperative for electric device applications in biological sensing.

## Figures and Tables

**Table 1:** Comparison of the electrical parameters of the ZnO-based TFT sensor under different conditions such as initial state and the various solvents on the exposed ZnO channel.

Parameters	Dielectric Constant	I_ON/OFF_	V_th_ (V)	µ_FE_ (cm^2^·V^−1^·s^−1^)	V_th_ (V)-After Drying
**Initial state**	-	~10^6^	2.75 ± 2.86	0.0053 ± 0.0012	-
**Toluene**	2.4	~10^7^	4	0.0082	5
**Ethanol**	24	~10^8^	−6	0.0014	1
**DI water**	80	Short circuit	Short circuit	Short circuit	5

## Supplementary Materials

The following are available online at www.mdpi.com/1996-1944/10/3/234/s1, Figure S1: Transfer characteristic of the square root of drain current plotted on a linear scale of solution-processed ZnO-based TFT sensors for a non-polar liquid solvent of toluene with a dielectric constant value of approximately 2.4, Figure S2: Transfer characteristic of the square root of drain current plotted on a linear scale of solution-processed ZnO-based TFT sensors for a polar liquid solvent of ethanol with a dielectric constant value of approximately 24, Figure S3: Transfer characteristic of the square root of drain current plotted on a linear scale of solution-processed ZnO-based TFT sensors for a DI water with high dielectric constant of approximately 80, leading to a highly charged surface on the channel surface, Figure S4: The thickness of ZnO channel film used was approximately 22 nm prepared by the spin-coating.
