# Supplementary material for: Low-Temperature, Solution-Processed, Transparent Zinc Oxide-Based Thin-Film Transistors for Sensing Various Solvents
Source: Materials (Basel). 2017 Feb 26;10(3):234. doi: 10.3390/ma10030234 (PMC5503344; doi:10.3390/ma10030234)
Supplement: Supplementary file 1 [file materials-10-00234-s001.pdf]

Supplementary Materials: Low-Temperature, Solution-Processed, Transparent Zinc Oxide-Based Thin-Film Transistors for Sensing Various Solvents

Hsin-Chiang You and Cheng-Jyun Wang

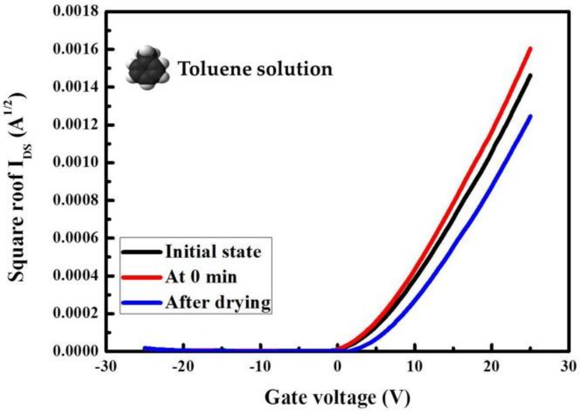

Figure S1. Transfer characteristic of the square root of drain current plotted on a linear scale of solution-processed ZnO-based Thin Film Transistor sensors for a non-polar liquid solvent of toluene with a dielectric constant value of approximately 2.4.

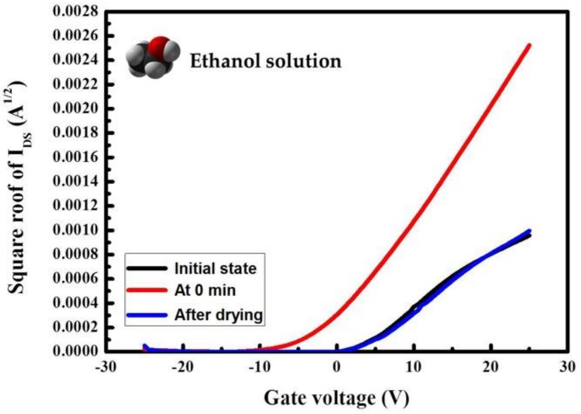

Figure S2. Transfer characteristic of the square root of drain current plotted on a linear scale of solution-processed ZnO-based TFT sensors for a polar liquid solvent of ethanol with a dielectric constant value of approximately 24.

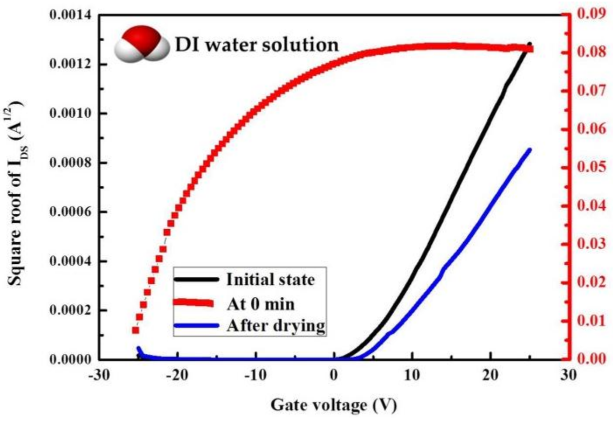

Figure S3. Transfer characteristic of the square root of drain current plotted on a linear scale of solution-processed ZnO-based TFT sensors for a DI water with high dielectric constant of approximately 80, leading to a highly charged surface on the channel surface.

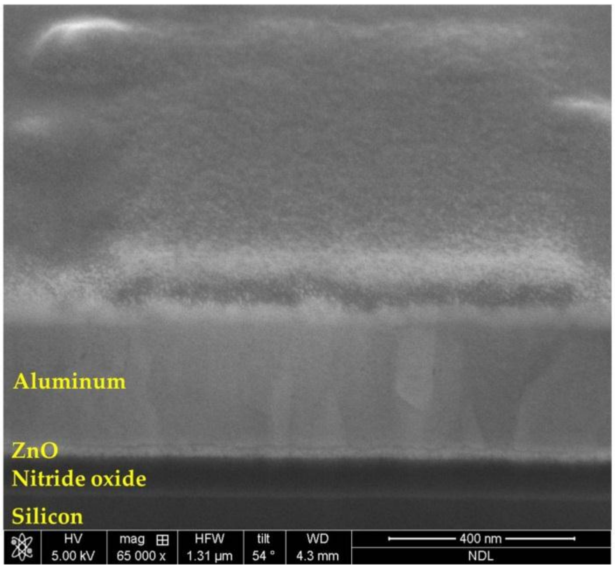

Figure S4. The thickness of ZnO channel film used was approximately 22 nm prepared by the spin-coating.

© 2017 by the authors; licensee MDPI, Basel, Switzerland. This article is an open access article distributed under the terms and conditions of the Creative Commons Attribution (CC BY) license (http://creativecommons.org/licenses/by/4.0/).
